# Low temperature co-fired ceramic packaging of CMOS capacitive sensor chip towards cell viability monitoring

**DOI:** 10.3762/bjnano.7.179

**Published:** 2016-11-29

**Authors:** Niina Halonen, Joni Kilpijärvi, Maciej Sobocinski, Timir Datta-Chaudhuri, Antti Hassinen, Someshekar B Prakash, Peter Möller, Pamela Abshire, Sakari Kellokumpu, Anita Lloyd Spetz

**Affiliations:** 1 Microelectronics Research Unit, Faculty of Information Technology and Electrical Engineering, P.O.Box 4500, FI-90014 University of Oulu, Finland; 2 Department of Electrical & Computer Engineering and the Institute for Systems Research, University of Maryland, College Park, MD 20742, USA; 3 Faculty of Biochemistry and Molecular Medicine, University of Oulu, P.O. Box 5400, FI-90014 University of Oulu, Finland; 4 Advanced Design Organization, Intel Corporation, Hillsboro, USA; 5 Division of Applied Sensor Science, Department of Physics, Chemistry and Biology, Linköping University, SE-58183 Linköping, Sweden

**Keywords:** capacitance sensing, cell viability, lab-on-a-chip, low temperature co-fired ceramic (LTCC)

## Abstract

Cell viability monitoring is an important part of biosafety evaluation for the detection of toxic effects on cells caused by nanomaterials, preferably by label-free, noninvasive, fast, and cost effective methods. These requirements can be met by monitoring cell viability with a capacitance-sensing integrated circuit (IC) microchip. The capacitance provides a measurement of the surface attachment of adherent cells as an indication of their health status. However, the moist, warm, and corrosive biological environment requires reliable packaging of the sensor chip. In this work, a second generation of low temperature co-fired ceramic (LTCC) technology was combined with flip-chip bonding to provide a durable package compatible with cell culture. The LTCC-packaged sensor chip was integrated with a printed circuit board, data acquisition device, and measurement-controlling software. The packaged sensor chip functioned well in the presence of cell medium and cells, with output voltages depending on the medium above the capacitors. Moreover, the manufacturing of microfluidic channels in the LTCC package was demonstrated.

## Introduction

Biosafety regulations require ethical, simple, rapid, and cost effective methods for evaluating cytotoxicity, both short and long term. Traditional in vitro cytotoxicity evaluation methods include cell cultivation and label-based assay kits, which are often expensive and time-consuming end-point measurements. Furthermore, the labelling techniques used for cell viability screening are lethal to the cells. Hence there is a growing interest in noninvasive, label-free, real-time, data-rich biosensing systems that measure electrical, optical, magnetic, or mass related properties of the biological sample. Such sensing techniques include surface plasmon resonance spectroscopy [1], electrochemical quartz crystal microbalance measurements [2], optical sensing [3], impedimetric sensing [4–6], and capacitive sensing [7–11].

The lab-on-a-chip (LoC) concept is an excellent way to implement label-free, noninvasive, cost-effective cytotoxicity assessment. LoCs are miniaturized analytical tools that combine sophisticated microfluidics with sensing or analysis [12–14]. Lab-on-CMOS (LoCMOS) is an emerging class of LoC that combines LoC with integrated circuits (ICs). LOCs are often used for analyzing chemical or biological samples. However, when the wet world of biology meets the dry world of electronics, the technical challenge arises to build a package for the LoCMOS device that is able to withstand the hostile biological environment, which may include high temperature, humidity, and corrosive liquids (mammalian cells typically require 37 °C, >95% humidity, and a salt-containing medium for growth).

Low temperature co-fired ceramic (LTCC) technology in combination with flip-chip bonding is one method of producing durable, biocompatible packaging for LoCMOS devices. The advantage of the LTCC technology is the possibility of fast and simple 3D processing of ceramic material, and the possibility to integrate advanced functionality like buried active or passive components, heat sinks, sensors, actuators, microchannels, and energy harvesters in the package in one firing step during the processing [15]. The LTCC is tailor-made from multiple layers containing the printed components; the layers are laminated and sintered to form a 3D block. Since the previous versions of LTCC produced devices with toxic properties in biological applications, it has not really been considered in this area until recently [16–21]. For example, Luo and Eitel reported a LTCC material as a substrate for biosensors that is regarded as biocompatible [22]. Also, from our experience, cell growth, at least over 24 h, seems to be fully compatible with the LTCC material [23]. We suggest that the previous statement about LTCC material being non-biocompatible was probably made too hastily based on our current knowledge of the LTCC material.

We recently reported on an LTCC package that was flip-chip bonded to a complementary metal-oxide semiconductor (CMOS) integrated circuit (IC) chip to form a LoCMOS system [23]. It was designed with a CMOS chip for capacitance sensing and the intention is to develop a method for nanoparticle exposure of cells to establish cytotoxicity assessment of nanomaterials.

Capacitance measurements reflect the surface attachment of adherent cells. While healthy cells attach to the cultivation surface and spread out, dying cells ball up and eventually detach from the substrate. Therefore, the strength of the coupling as well as the area of the sensor surface covered by cells, measured by the capacitance of the chip, is an indication of cell viability. Capacitive sensing of a cell population on the chip is label-free, noninvasive, fast, and continuous.

Preliminary testing of the first generation LTCC package was performed using human epithelial cells cultivated on the chip. A very short, 3 h in total, trial measurement was performed and a small response related to sedimentation of cells on the chip was reported. Since the cell proliferation thus seemed to be normal, the use of the LTCC package for the sensor chip was regarded as promising [23].

Here we have tested version 2 of the LTCC package made from Dupont^TM^ 951 LTCC tape instead of the Heraeus HeraLock^®^ Tape HL2000 (no longer produced) used in our earlier version [23]. Human lung epithelial cells (BEAS2B) were cultivated on the chip to verify the biocompatibility of the package material. Responses were obtained on dry chips, chips covered with only cell growth medium (DMEM), and chips covered with a solution of cells in DMEM, demonstrating the robustness and functionality of the package. In addition, integration of microfluidic channels in the package was demonstrated.

## Experimental

### Dummy chips

**Figure 1:**
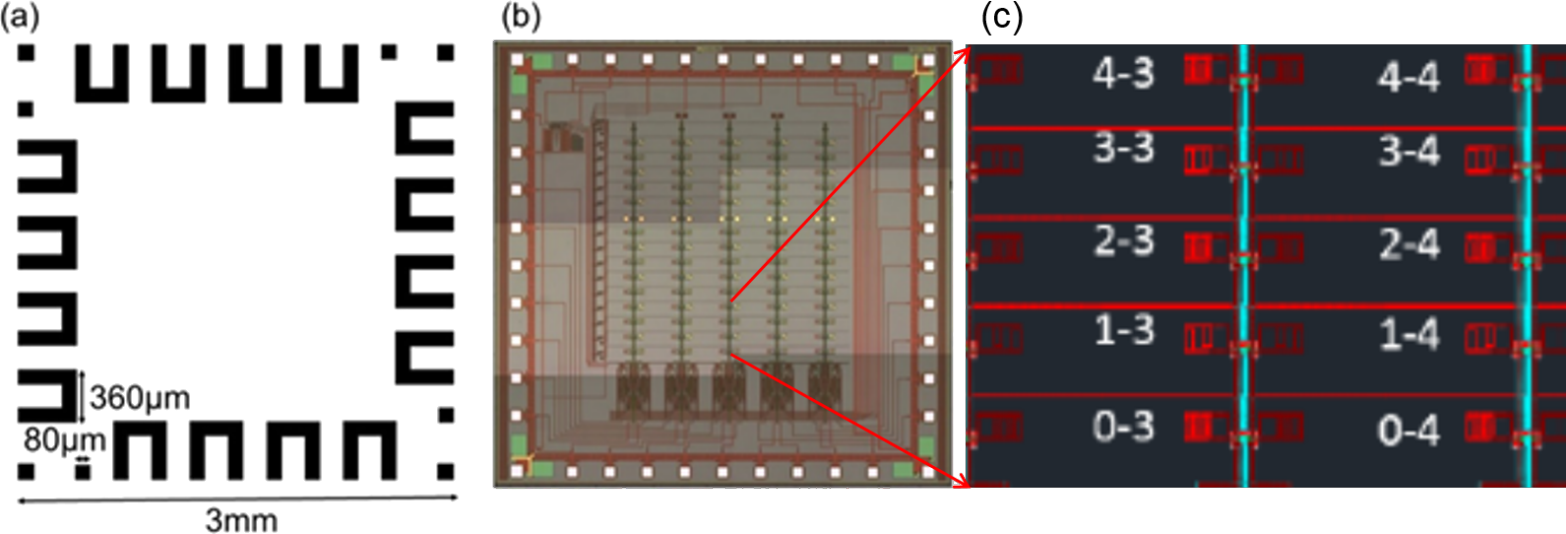
(a) Layout of the dummy chip mimicking the size of the sensor chip and the contact pad positions and sizes. (b) Microscope image of the capacitance sensor chip showing contact pads at the periphery and an array of sensors distributed over 16 rows and 5 columns. (c) Close up of capacitive finger electrode structures.

Dummy chips for testing purposes were fabricated on a 4 in silicon wafer (p++ type, boron-doped, resistivity <0.005 Ω·cm) with 500 nm of SiO_2_ (PECVD grown). Contact pads in the form of a U-shaped loop mimicked the location and size of the contact pads on the sensor chip. They consisted of RF sputtered gold (300 nm with 10 nm of chromium as adhesion layer, deposited by e-beam) (Figure 1a). The wafer was diced into 3 × 3 mm^2^ chips by laser scribing.

### Capacitance sensor chip

The capacitance sensor chips (3 × 3 mm^2^) were fabricated in a commercial 2-poly, 3-metal, 0.5 µm CMOS process, as demonstrated in Figure 1b and c. The fully differential sensor chip was designed for measuring capacitance in the ±25 fF range. Each sensor contained two interdigitated capacitors, one reference and one test capacitor for differential measurements, and four minimum-sized transistors, allowing the sensors to be packed densely. Cells located over the interdigitated plates of the capacitors increase the effective capacitance. The sensor array consisted of 16 rows and 5 columns. Within each pixel, charge accumulated on the capacitors. The four transistors acted as switches to: 1) reset the pixel voltage between measurements and 2) select the desired row for readout. The readout circuit incorporated a floating gate transistor that allowed compensation for fabrication mismatch [10]. The chip had 40 Al/TiN/Cu contact pads with a size of 85 µm and a 120 µm spacing. Because oxidation of the Al in the pads at the rim of the chip prevented the bonding of the chip to the LTCC package with conductive adhesive, gold bumps were applied onto the pads with a gold wire (20 µm in diameter) bonder. The wire bonding process improved the electrical contact between the chip and the LTCC package, presumably by punching through the Al oxide. The gold wires were then manually removed, leaving a gold bump on the contact pads for the following steps.

#### LTCC package

Commercial Dupont 951 Green Tape^TM^ LTCC was used to fabricate packages for the sensor chip. On the tape, the conductor lines were printed with Dupont 6142D silver co-fireable conductor paste. The recommended standard LTCC process (data sheet from manufacturer) was used to manufacture and fire the package, while a special process, described below, was adapted for the microfluidic channels.

The sensor chips were glued to the LTCC packages with isotropic conductive adhesive (ICA) (EPO-TEK, H20E-PFC). The adhesive consisted of conductive silver particles embedded in adhesive polymer resins. This two-component epoxy was chosen for its ability to form small-sized patterns to connect the closely spaced contact pads.

**Figure 2:**
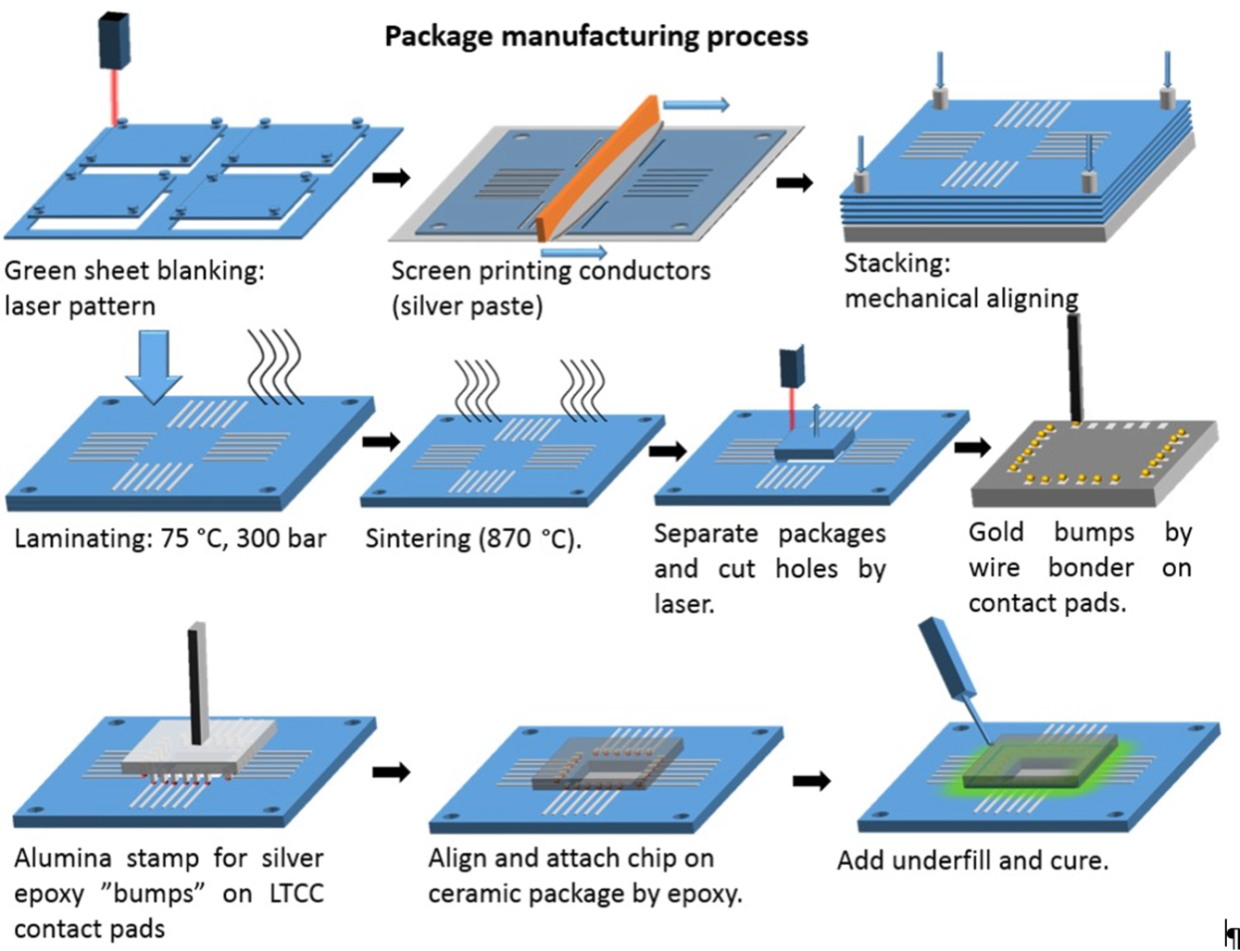
Schematic image of the isotropic conductive adhesive stamping process, sensor chip mounting, and underfill application.

The ICA was applied on the contact pads of the LTCC as “bumps” with a stamping process (Figure 2). The stamp was made of alumina by laser processing. The sensor chip was glued onto the bumps by epoxy and cured at 150 °C on a hot plate. For alignment a flip-chip bonder was used. An epoxy underfill (EPO-TEK, 302-3M) was applied around the bumps and cured at 65 °C, to provide a seal between the chip and the LTCC against the liquid as well as additional attachment strength. To hold the fluid over the sensor surface, the same underfill material was used to glue a well on top of the LTCC package. Furthermore, a nonstandard process was used to manufacture a package with an integrated microfluidic channel [24]. Fluidic channels were manufactured using special lamination technique involving lower pressure (150 bar, 15 min) and sacrificial carbon tape (C12, Advanced Technologies). The intention was to attach suitable tubes between the fluidic channels and a reservoir and to use, for example, a peristaltic pump to transport the liquid to the sensor chip.

**Figure 3:**
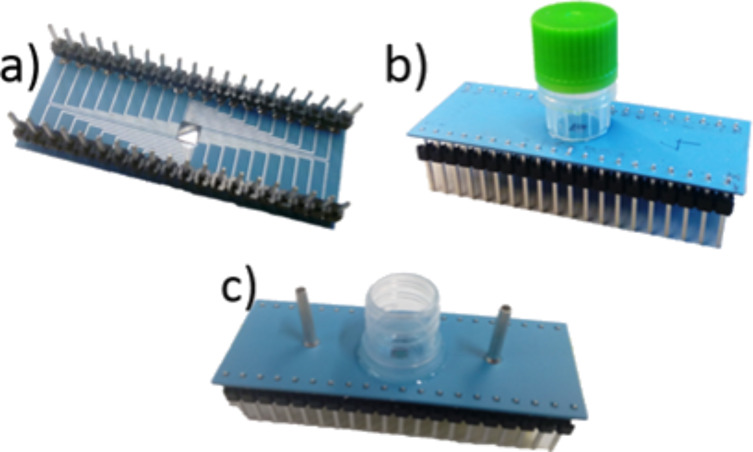
(a) Bottom side of the LTCC package showing the rear side of the sensor chip. (b) The sensor chip in the LTCC package connected. The active side of the chip is inside the cell culture vial glued on the top of the LTCC package. (c) Prototype of the package with integrated microfluidic channel.

The packaged chip was connected, using a zero insertion force (ZIF) connector, to a printed circuit board (PCB). Figure 3a,b shows the manufactured package and Figure 3c the prototype package with microfluidic channel.

## Results and Discussion

### Reliability of the packaging method

The new LTCC material was first tested using a dummy chip packaged in the LTCC and connected to a PCB that linked the pads in a daisy chain pattern. Two packaged dummy chips with wells filled with cell medium were placed in a cell incubator for 8 days (37 °C, 5% CO_2_, 95% air). The total resistance of the package was monitored by a two-point measurement using a digital multimeter. On the 6th day, BEAS2B cells were added onto the chip.

**Figure 4:**
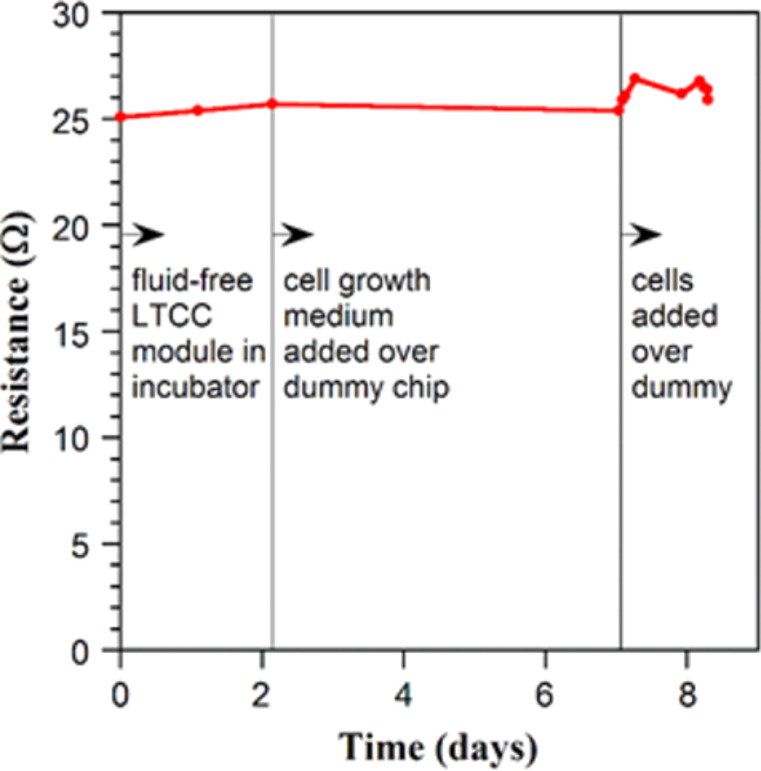
The total resistance of an LTCC packaged dummy chip placed in a cell culture incubator before and after cell growth media and BEAS2B cells were added onto the chip.

Figure 4 shows the total resistance of the first dummy chip in the cell cultivation environment; the second one showed similar behavior. The average of the total resistance over the bond pads in series was 26 ± 0.6 Ω during the measurement period, showing that the bonds were electrically and mechanically stable and protected from the cell medium. The resistance increased by a few ohms when the cells were added on the chip. When taking the package out of the incubator (the last part of the curve in Figure 4), the resistance value returned to the original level possibly due to mechanical disturbance caused by the procedure.

### Biocompatibility of the LTCC package

**Figure 5:**
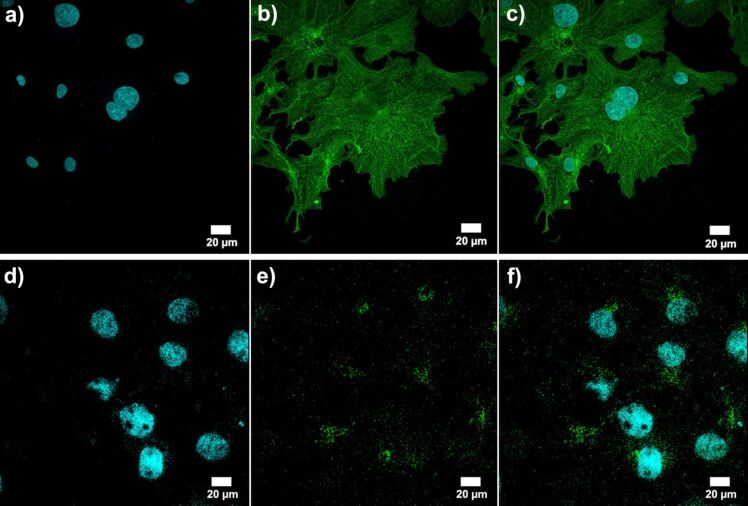
Normally proliferating BEAS2B cells on a dummy chip in LTCC package. (a–c) the cells grow on top of the chip. (d–f) The cells grow on top of LTCC. In (a) and (d), the blue color indicates the cell nuclei stained with a DNA binding dye, Hoechst 33342. In (b) and (e), immunofluorescence staining was performed with anti-α-tubulin antibody and Alexa 488 secondary antibody. The green color shows the microtubules of the cell cytoskeleton. In (c) and (f), the merged image of the nuclear staining and cytoskeleton are shown. The images were taken with a Zeiss LSM700 confocal microscope with 63× plan-apo immersion objective and appropriate filter sets.

The earlier reported [23] biocompatibility of the LTCC package to cell culture was also confirmed here for the new LTCC material by growing BEAS2B cells on a dummy chip and on the surrounding LTCC. The attachment of the cells on the chip, as well as LTCC, was monitored by fixing the cells with 4% paraformaldehyde 24 h after inoculation and staining the cells with a DNA binding dye (Hoechst, 33342) and anti-α-tubulin antibody. Based on the cell morphology shown in Figure 5a–f, the cells attached normally and spread out over the surface of the chip and LTCC. The Dupont 951 LTCC material has also been reported as biocompatible by others [25].

### Cell measurements

To demonstrate the feasibility of using the LTCC package in biosensing applications, data were recorded for several hours from a CMOS sensor chip in the package. The cell measurement set up included an LTCC module and a printed circuit board (PCB), placed inside the incubator, and a data acquisition system (National Instruments, NI-USB 6259) connected to a computer running Matlab-based control software, placed outside the incubator.

Prior to the measurements, the outputs of the capacitance sensors were adjusted using the custom software until they reached an initial target value of approximately 1.5 V. This procedure centered the sensor response within the power and ground voltage range, allowing variations in both directions from the baseline to be recorded.

**Figure 6:**
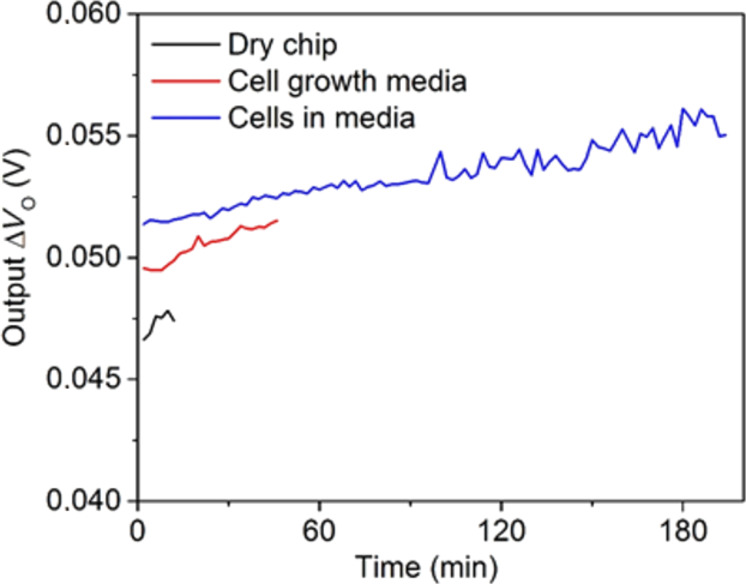
Average voltage change from the baseline over time from all sensors on one chip after cell media and cells were added. The average signal from the sensors on the dry chip is added for comparison.

A sensor chip with no fluid in the well was placed inside the incubator and data was recorded for 12 min. Then 300 µL of cell growth medium (DMEM) was added into the well, and data was recorded for 50 min with data points taken every 2 min. The maximum rate of the system was 60 frames/min. The CMOS chip continued to function upon the addition of the fluid, confirming the robustness of the package. The important thing to note is that the signal increased upon the addition of DMEM (Figure 6). This was expected because the dielectric constant of water is higher than that of air, resulting in a higher capacitance between the electrodes. The chip returned to its original level after the liquid was removed (not shown). Finally, cells were added to the surface of the chip in growth medium, and data was recorded for 3 hours. Again the CMOS chip continued to function upon the addition of cells. The capacitance signal again increased with the addition of cells, which is consistent with expectations and prior work [7,9].

## Conclusion

A commercial LTCC material was used to package CMOS sensor chips. LTCC provides the possibility to integrate new functions into Lab-on-CMOS packages, and the integration of microfluidics into the package was demonstrated. Normal cell morphology on packaged dummy chips demonstrated the feasibility of using the LTCC package for cell culture; no cytotoxicity was observed. Furthermore, it was possible to obtain sensor measurements in real time. The capacitance varied abruptly as the overlying medium changed, demonstrating that the package and chip were communicating successfully. Future developments will include applications such as monitoring the influence on cell viability of nanomaterials or drugs.
